# Inferior Pole Adipose Dermal Flap for Breast Reconstruction: A Novel Oncoplastic Technique for Breast-Conserving Surgery

**DOI:** 10.1093/asjof/ojaf096

**Published:** 2025-07-23

**Authors:** Francesco Klinger, Riccardo Di Giuli, Alessandra Veronesi, Barbara Catania, Stefano Vaccari, Valeria Bandi, Marco Klinger, Valeriano Vinci

## Abstract

**Background:**

Breast cancer is a prevalent malignancy among women, and advancements in treatment have shifted focus to enhancing both survival and quality of life. Oncoplastic surgery integrates oncological safety with aesthetic considerations.

**Objectives:**

The authors of this study aim to evaluate the efficacy of the inferior pole adipose dermal (IPAD) flap for lower pole reconstruction in breast-conserving surgery (BCS).

**Methods:**

A retrospective cohort study was conducted involving patients who underwent BCS with IPAD flap reconstruction. Inclusion criteria comprised female patients over 18 years with at least 12 months of follow-up. Data collected included demographics, clinical history, surgical details, complications, and patient satisfaction, assessed using the Visual Analog Scale (VAS).

**Results:**

The study included 12 patients with a mean age of 52.7 years and a mean BMI of 28. Four patients (33.3%) required integration of a perforator flap for additional volume. No major complications, such as infections or flap necrosis, were observed. Minor complications included lower pole retraction (16.6%) and inferior dehiscence (8.3%). Patient satisfaction was high (VAS 7.5), and aesthetic outcomes were rated positively by surgeons (7.2). Adjuvant radiotherapy did not adversely affect flap viability.

**Conclusions:**

The IPAD flap may be a viable and effective technique for lower pole reconstruction in BCS. This technique is particularly beneficial for patients with adequate breast volume. The authors of this study underscore the importance of personalized surgical planning and a multidisciplinary approach to optimizing breast cancer treatment.

**Level of Evidence: 4 (Therapeutic):**

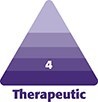

Breast cancer remains one of the most common malignancies affecting women worldwide, with a significant impact on morbidity and mortality. According to the World Health Organization, ∼2.3 million women were diagnosed with breast cancer in 2022, with 670,000 deaths globally.^1^ Advances in early detection and treatment have improved survival rates, shifting the focus toward not only survival but also the quality of life posttreatment.^2^ This evolution in breast cancer care has highlighted the importance of breast-conserving surgery (BCS), a procedure aimed at excising the tumor while preserving as much of the breast tissue as possible.

BCS has advanced considerably because it was first implemented. Initially, the primary goal was to achieve oncological safety, ensuring complete tumor removal with clear margins. Over time, however, the aesthetic outcomes of BCS have become equally important. The development of oncoplastic surgery, which integrates principles of oncologic surgery with plastic surgery techniques such as the periareolar approach, has allowed for more extensive resections without compromising cosmetic outcomes.^3,4^ This strategy has proven to be particularly beneficial for patients with larger tumors where traditional lumpectomy might lead to significant deformities.^5^

Breast remodeling techniques have become an essential component of oncoplastic surgery.^6,7^ The utilization of local glandular flaps, for instance, provides a reliable method to fill the defect created by the tumor removal while ensuring that the breast retains a natural appearance. The inferior pole adipose dermal (IPAD) flap involves mobilizing inferior breast tissue based on a superior-based vascular pedicle, ensuring robust blood supply and tissue viability for breast reconstruction. Derived from an aesthetic technique used in reduction mammoplasty to enhance the lower pole fullness, this method employs the inferior breast section to create versatile and reliable flaps for addressing inferior defects.^8^ When the resection is extensive, reconstruction may benefit from combining breast remodeling with autologous volume replacement to achieve optimal cosmetic and functional outcomes.^9^

In this paper, we present our experience with the IPAD flap in BCS. We describe the surgical technique and its application, presenting our clinical and surgical outcomes.

## METHODS

### Study Design

In this study, we conducted a retrospective analysis of patients who underwent BCS and reconstruction using the IPAD flap technique in Humanitas Research Hospital from January 2021 to June 2023. The inclusion criteria encompassed female patients older than 18 years who underwent primary BCS procedures involving the IPAD flap for lower pole reconstruction, with a mandatory follow-up period of at least 12 months. Exclusion criteria included noncompliance with follow-up appointments and the absence of documented information. All procedures involving human participants in the studies were conducted in compliance with the ethical standards of the institutional and/or national research committees, as well as the 1964 Declaration of Helsinki and its subsequent amendments or comparable ethical guidelines. Informed consent for the procedure was obtained from all patients before surgery during the preoperative visit.

The collected data from participants included demographics (age, BMI), clinical history, surgical technical details, and any encountered complications. Postoperative complications were classified according to the Singhal classification system for complications in plastic and reconstructive surgery.^10^ Additionally, photographic documentation was systematically collected, capturing preoperative and postoperative images at every follow-up visit to facilitate a comprehensive reassessment of patients. Patient satisfaction was evaluated at 1-year follow-up using the Visual Analog Scale (VAS), and aesthetic outcomes were assessed by 2 independent surgeons on a scale from 1 to 10.

### Surgical Technique

Preoperative markings were planned in collaboration with the breast surgeon to access the breast gland through either an inframammary fold or Wise pattern incision. Following the excision of the tumor and a margin of healthy tissue, the tissue located in the inferior horizontal section of the breast was de-epithelialized and mobilized through the inframammary incision while maintaining a superior pedicle. Inferior pole resections may be addressed solely through the inframammary approach, utilizing de-epithelialized inferior pole breast tissue and subcutaneous upper thoracic tissue.

**Figure 1.**
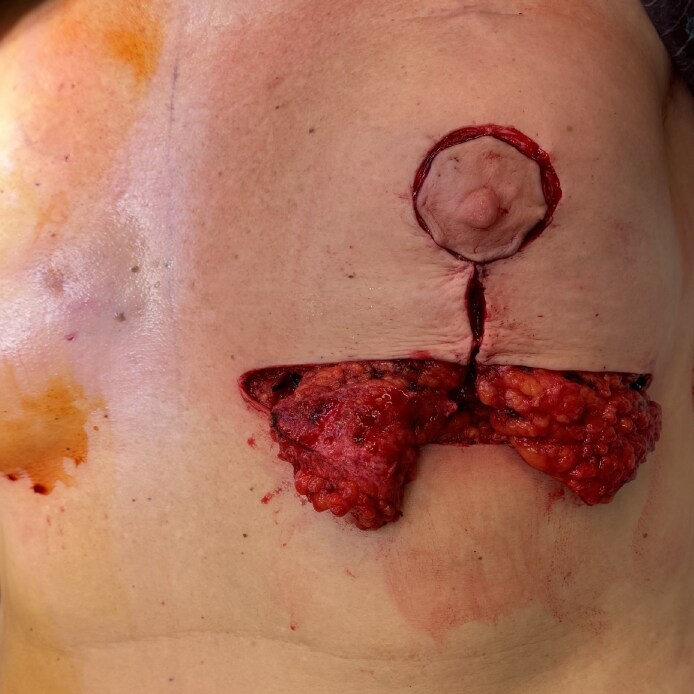
Intraoperative view of the interior of the left breast (inferior pole adipose dermal) flaps of a 52-year-old female patient after inferior pole de-epithelialization and flap dissection. Central lower pole tumor resection was previously performed through the vertical access of a Wise pattern incision.

**Figure 2.**
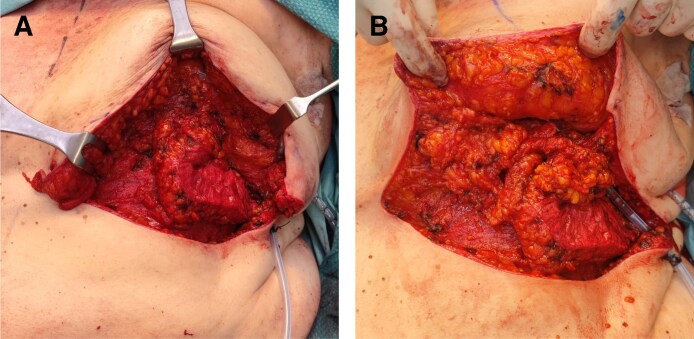
(A) Intraoperative view of the anterior of the left breast intercostal perforator flap translocation for partial defect reconstruction in a 63-year-old female patient. (B) Inferior pole adipodermal flaps were dissected and mobilized to provide additional tissue to the perforator flap.

The glandular tissue was carefully dissected from the fascial plane, and a deliberate dermal incision at the flap base was performed to facilitate its rotation and transposition, as illustrated in Figure 1. Based on tumor localization, either 1 or 2 flaps may be mobilized. Two flaps are available for central defects, whereas a single flap is employed for lateral defects. Precise insetting was performed to preserve the natural contour of the breast and achieve optimal lower pole fullness. The superior pedicle, coupled with inferior dissection, provides extensive mobilization potential. These flaps are translocated medially and rotated internally, allowing for placement either adjacent to each other or overlapped to enhance projection and fixated to the surrounding residual glandular tissue employing Vicryl (polyglactin 910; Ethicon, Raritan, NJ). In cases of larger defects, an intercostal perforator flap was dissected and mobilized superiorly to provide additional tissue for reconstructing the glandular defect (Figure 2, Video). Previous identification of perforators was conducted using a handheld Doppler in the territory of the pertinent intercostal artery for large resections. Incisions were closed in layers before the placement of a drain, using Vicryl (polyglactin 910, Ethicon) for the deep tissues and Monocryl (poliglecaprone 25, Ethicon) in a continuous intradermal fashion to optimize aesthetic outcomes and minimize scarring. Particular attention was given to reconstructing the inframammary sulcus when a perforator flap was utilized. A sterile dressing was applied over the skin, followed by a mild elastocompressive bandage.

Patients received preoperative intravenous antibiotic prophylaxis with 2 g of cefazolin or 600 mg of clindamycin if allergic. No prophylactic postoperative antibiotics were administered. Postoperative care included monitoring patients for complications such as infection, hematoma, flap necrosis, or dehiscence. Assessment for fat necrosis was performed exclusively through clinical examination during these follow-up visits. One week after surgery, the compressive bandage and drain were removed, and a supportive surgical bra was applied. Follow-up visits were scheduled weekly for the first month, then at 3 months, 6 months, and annually to assess healing, aesthetic outcomes, and patient satisfaction. Photographic documentation was obtained at each visit to systematically track progress and outcomes.

## RESULTS

Twelve female patients met the inclusion and exclusion criteria. Clinical observations were documented during preoperative visits. The mean age was 52.7 years (range, 38-68 years; standard deviation [SD] 8.8), and the mean BMI reported was 28 (range, 24.4-32; SD 2.9; Table 1). Detailed information regarding tumor characteristics is provided in Supplemental Table 1. No major comorbidities were identified, with 2 patients (16.6%) having hypertension and 1 patient (8.3%) having diabetes. The minimum follow-up period was 12 months, with an average follow-up of 21 months (range, 12-38 months). Isolated IPAD flap reconstruction was performed on 8 patients (66.6%), whereas 4 patients (33%) required an anterior intercostal perforator flap in addition to the flaps to reconstruct the lower pole. Nine patients (75%) underwent simultaneous contralateral breast symmetrization.

**Table 1.** Patient Data for Inferior Pole Dermal Adipose Flap Reconstruction

Patients	Age	BMI	Smoking habit	Breast ptosis (Regnault classification)	Follow-up (months)	Radiotherapy	Hematoma	Flap necrosis	Wound dehiscence	Lower pole retraction	Secondary revision	Autologous flap integration	Contralateral breast symmetrization
P1	63	31.3	No	2	24	No	No	No	No	Yes	No	Yes	No
P2	52	28.2	No	1	14	Yes	No	No	No	No	No	No	Yes
P3	48	25.7	No	2	30	No	No	No	No	No	No	Yes	Yes
P4	38	27.0	No 2	22	No	No	No	No	No	No	No	Yes	
P5	45	24.4	Yes	3	12	No	No	No	No	No	No	No	Yes
P6	59	32.0	No	3	38	Yes	No	No	Yes	No	No	No	Yes
P7	61	31.4	No	3	21	No	No	No	No	No	No	No	Yes
P8	52	27.0	No	2	13	Yes	No	No	No	No	No	No	Yes
P9	55	25.1	Yes	2	15	No	No	No	No	Yes	Yes (lipofilling)	No	Yes
P10	47	25.3	No	2	26	Yes	No	No	No	No	No	Yes	No
P11	68	32.0	No	3	18	Yes	No	No	No	No	No	No	Yes
P12	44	26.7	Yes	2	19	No	No	No	No	No	No	Yes	No

No major complications, such as infection, were reported (Table 1). During follow-up visits, lower pole retraction was observed in 2 cases (16.6%). In one of these cases, a lipofilling procedure was performed, which was the only instance requiring reoperation. The other case of retraction was minor and did not require further surgical intervention. One patient (8.3%) underwent a mastectomy due to tumor recurrence 26 months after partial breast resection. Additionally, 1 patient (8.3%) developed inferior cutaneous dehiscence 2 weeks postsurgery, which healed completely without complications following conservative management with local wound care and dressings. Five patients (41.7%) received adjuvant radiotherapy.

**Figure 3.**
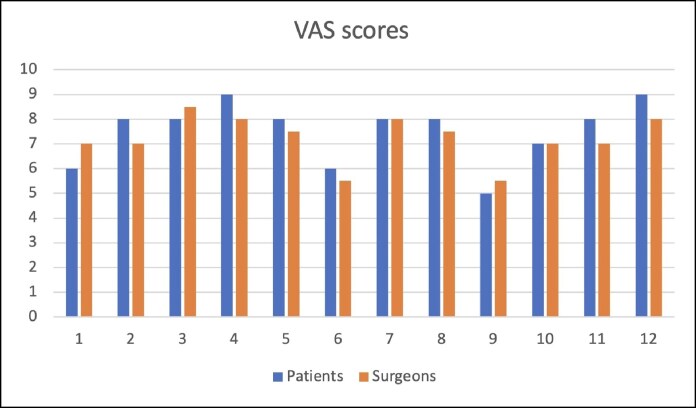
Visual Analog Scale scores for assessment of aesthetic outcome at 12 months postsurgery.

The VAS administered at 12 months postsurgery reported high levels of satisfaction by the patients (7.5; range, 5-9). Similarly, the scores provided by the surgeons also indicated favorable outcomes (7.2; range, 5.5-8.5; Figure 3).

## DISCUSSION

In this study, the authors provide evidence supporting the efficacy and safety of the IPAD flap for lower pole reconstruction in BCS, suggesting potential advantages in terms of aesthetic outcomes and patient satisfaction. The inframammary fold or Wise pattern access design facilitates high utilization rates and underscores the versatility of this technique. One notable aspect of our technique is the incision of the dermis, which, contrary to concerns, does not compromise the integrity or viability of the flap. The dermis can be safely incised to increase the mobilization and transposition of the tissue.^4,11^ This approach allows for greater flexibility and precision in reshaping the breast, ensuring that the reconstructed breast retains a natural contour and symmetry.

The utilization of a superior-based vascular pedicle in inferior flaps ensures robust blood supply and reliable tissue viability, which is critical for the success of breast reconstructions. The IPAD flap's vascular reliability is further supported by anatomical studies describing consistent perfusion of the lower and central breast through perforating branches of the anterior intercostal arteries, arising from the middle portion of the breast, as well as internal mammary perforators and the subdermal plexus.^12-14^ These vascular networks remain preserved during the flap mobilization and allow for safe transposition when tension-free fixation is achieved.

Horizontally, the flap can extend across the full width of the breast base, although adjustments such as partial lateral base resection are often employed to facilitate optimal insetting and contouring. Vertically, the anatomical boundaries for flap harvest have not been definitively established and appear to vary with individual patient morphology; nonetheless, our current strategy emphasizes achieving a tension-free closure of the inframammary fold. With regard to flap thickness, full-thickness harvesting of the glandular tissue in the inferior pole is advocated to enhance vascularization and ensure sufficient volume for reconstruction.

Each patient presents unique challenges and anatomical considerations, and a one-size-fits-all approach is not adequate. Our findings indicate that this technique is particularly beneficial for patients with ptotic breasts with at least moderate breast hypertrophy. In such cases, the availability of tissue allows for effective breast remodeling. The presence of glandular tissue often enables customization of the flap volume and shape, including the excision of lateral flap components, to achieve improved roundness of the lower pole. In patients with smaller breasts, the reduced initial breast volume does not permit the utilization of these flaps for inferior lower pole reconstruction, thereby constituting a limitation in the application of this technique. Additionally, patients with minimal vertical height in the lower pole region are generally not ideal candidates, as there is an increased risk of excessively low nipple positioning following reconstruction. Instead, lower pole remodeling procedures or autologous reconstruction should be employed.^15-17^

The combination of breast remodeling and volume replacement highlights the importance of personalized surgical planning in BCS.^9^ Personalized planning allows for tailored surgical interventions that address both the oncologic and aesthetic needs of the patient. Several authors have reported various new techniques and applications of perforator flaps in partial breast reconstruction.^18-21^ In our cases, the utilization of perforator flaps provided the necessary tissue to complete reconstruction in cases of extensive lower pole resections, where the inferior flaps mobilization alone would not supply sufficient tissue (Video). In our experience, the anterior intercostal perforator flap is the preferred choice, as it allows dissection through the same inframammary incision and benefits from direct tissue continuity with the inferior flaps.

The flap mobilization enabled the provision of tissue to reconstruct the basal over-fascial breast, with IPAD flaps placed onlay to enhance the fullness of the inferior pole as described in the Video. Effective breast replacement reduces the need for additional surgeries on the contralateral breast to achieve symmetry, thereby minimizing surgical risks and preserving breast tissue.^17^

In our study, the utilization of the IPAD flap resulted in minimal complications. No major issues such as infections or flap necrosis were reported, and only a small percentage of patients experienced minor complications like lower pole retraction or inferior dehiscence. These complications were effectively managed with conservative treatments or minor secondary procedures, further underscoring the reliability of this technique.

The role of adjuvant radiotherapy in breast oncoplastic surgery is poorly studied in the literature.^18^ Despite the potential risks associated with radiation, our results showed that the inferior flap maintained its viability and effectiveness, even in patients who underwent radiotherapy.^22,23^ The only case requiring corrective lipofilling was not associated with radiotherapy. This finding suggests that our technique can be safely and effectively integrated into comprehensive breast cancer treatment plans that include radiotherapy.

Incorporating plastic surgical techniques into the oncologic setting underscores the importance of a multidisciplinary approach to breast cancer treatment. By combining oncologic safety with aesthetic considerations, we can significantly improve the quality of life for breast cancer patients.^24-26^

This technique has been developed based on aesthetic principles designed to enhance fullness in the inferior pole of a reduction mammoplasty, originally employing these flaps as an “auto-implant.”^8^

This approach recognizes that methods proven effective in aesthetic surgery can be successfully adapted to oncologic settings, achieving optimal outcomes. Although originally conceived in an aesthetic context, this concept has evolved into a highly versatile reconstructive technique, allowing for precise volume refinement of the lower.

**Figure 4.**
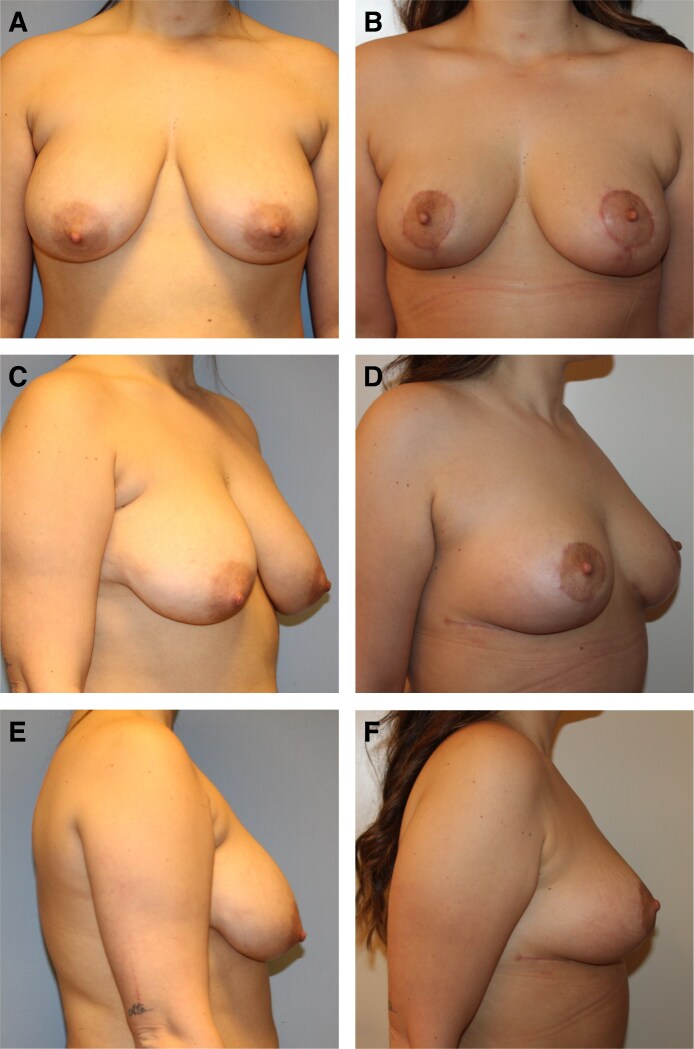
(A) Preoperative and (B) 6-month postoperative frontal view, (C) preoperative and (D) 6-month postoperative right-quarter view, (E) preoperative and (F) 6-month postoperative right lateral view of a 38-year-old female patient who underwent inferior right breast partial mastectomy and breast remodeling technique with inferior pole adipose dermal flap along with simultaneous contralateral breast symmetrization.

**Figure 5.**
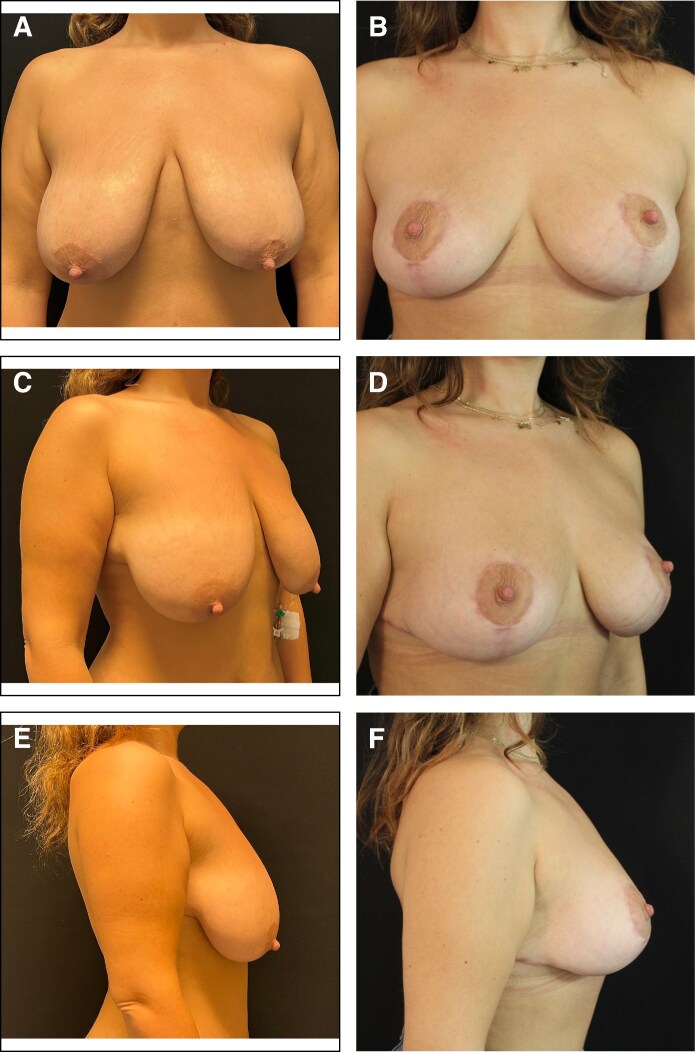
(A) Preoperative and (B) 6-month postoperative frontal view, (C) preoperative and (D) 6-month postoperative right-quarter view, (E) preoperative and (F) 6-month postoperative right lateral view of a 45-year-old female patient who underwent inferior right breast partial mastectomy and subsequent breast remodeling using the inferior pole adipose dermal flap technique, combined with simultaneous contralateral breast symmetrization.

Patients reported a high degree of satisfaction with the aesthetic outcomes of the oncoplastic surgeries using the IPAD flap (Figures 4, 5). Surgeons also indicated substantial satisfaction with the results, although slightly less so compared with the patients, suggesting minor areas for potential enhancement.

Our study has several limitations, including its retrospective design, relatively small sample size, and the subjective nature of the outcome assessment. Additionally, there was no quantitative assessment of the vascular supply of the IPAD flap, and fat necrosis was evaluated solely through clinical examination, without adjunctive imaging. Larger prospective studies with longer follow-up are needed to further validate our findings and refine the technique. However, the consistent positive outcomes observed in our cohort provide a strong foundation for the continued utilization and development of the IPAD flap in BCS.

## CONCLUSIONS

The IPAD flap may represent a promising addition to the reconstructive options available to oncoplastic breast surgeons. It aims to integrate oncologic safety with aesthetic considerations, potentially offering a comprehensive approach to BCS. The technique is designed to ensure adequate vascularity and tissue viability while allowing for individualized surgical planning. Although preliminary results appear encouraging, further studies are needed to confirm its reliability and effectiveness in achieving both oncologic and aesthetic goals.

## Supplemental Material

This article contains supplemental material located online at https://doi.org/10.1093/asjof/ojaf096.

## Supplementary Material

ojaf096_Supplementary_Data
