# Supplementary material for: Inferior Pole Adipose Dermal Flap for Breast Reconstruction: A Novel Oncoplastic Technique for Breast-Conserving Surgery
Source: Aesthet Surg J Open Forum. 2025 Jul 23;7:ojaf096. doi: 10.1093/asjof/ojaf096 (PMC12548049; doi:10.1093/asjof/ojaf096)
Supplement: ojaf096_Supplementary_Data [file ojaf096_Supplementary_Data.docx]

| **Supplementary Tab.1** Breast Tumors Characteristics | | | | | |
| --- | --- | --- | --- | --- | --- |
| Patients | T | N | M | Weight (g) | Molecular Phenotypes |
| P1 | T2 | 0 | 0 | 123 | Luminal B |
| P2 | T2 | 0 | 0 | 74 | Luminal A |
| P3 | T1 | 0 | 0 | 62 | Luminal A |
| P4 | T1 | 0 | 0 | 40 | Luminal A |
| P5 | T2 | 0 | 0 | 60 | Luminal A |
| P6 | T2 | 0 | 0 | 74 | Luminal B |
| P7 | T1 | 0 | 0 | 61 | Luminal A |
| P8 | T2 | 0 | 0 | 54 | Luminal A |
| P9 | T1 | 0 | 0 | 36 | Luminal A |
| P10 | T2 | 0 | 0 | 64 | Luminal A |
| P11 | T2 | 1 | 0 | 71 | Luminal B |
| P12 | T1 | 0 | 0 | 51 | Luminal A |
